# Digital reconstruction of the mandible of an adult *Lesothosaurus diagnosticus* with insight into the tooth replacement process and diet

**DOI:** 10.7717/peerj.3054

**Published:** 2017-03-01

**Authors:** Lara Sciscio, Fabien Knoll, Emese M. Bordy, Michiel O. de Kock, Ragna Redelstorff

**Affiliations:** 1 Department of Geological Sciences, University of Cape Town, Cape Town, South Africa; 2 Fundación Conjunto Paleontológico de Teruel-Dinópolis, Teruel, Spain; 3 Department of Geology, University of Johannesburg, Johannesburg, South Africa

**Keywords:** Early jurassic, Elliot formation, Gondwana, South Africa, *Lesothosaurus diagnosticus*, Ornithischia, micro-CT, Diet, 3D reconstruction

## Abstract

Fragmentary caudal ends of the left and right mandible assigned to *Lesothosaurus diagnosticus*, an early ornithischian, was recently discovered in the continental red bed succession of the upper Elliot Formation (Lower Jurassic) at Likhoele Mountain (Mafeteng District) in Lesotho. Using micro-CT scanning, this mandible could be digitally reconstructed in 3D. The replacement teeth within the better preserved (left) dentary were visualised. The computed tomography dataset suggests asynchronous tooth replacement in an individual identified as an adult on the basis of bone histology. Clear evidence for systematic wear facets created by attrition is lacking. The two most heavily worn teeth are only apically truncated. Our observations of this specimen as well as others do not support the high level of dental wear expected from the semi-arid palaeoenvironment in which *Lesothosaurus diagnosticus* lived. Accordingly, a facultative omnivorous lifestyle, where seasonality determined the availability, quality, and abundance of food is suggested. This would have allowed for adaptability to episodes of increased environmental stress.

## Introduction

**Figure 1:**
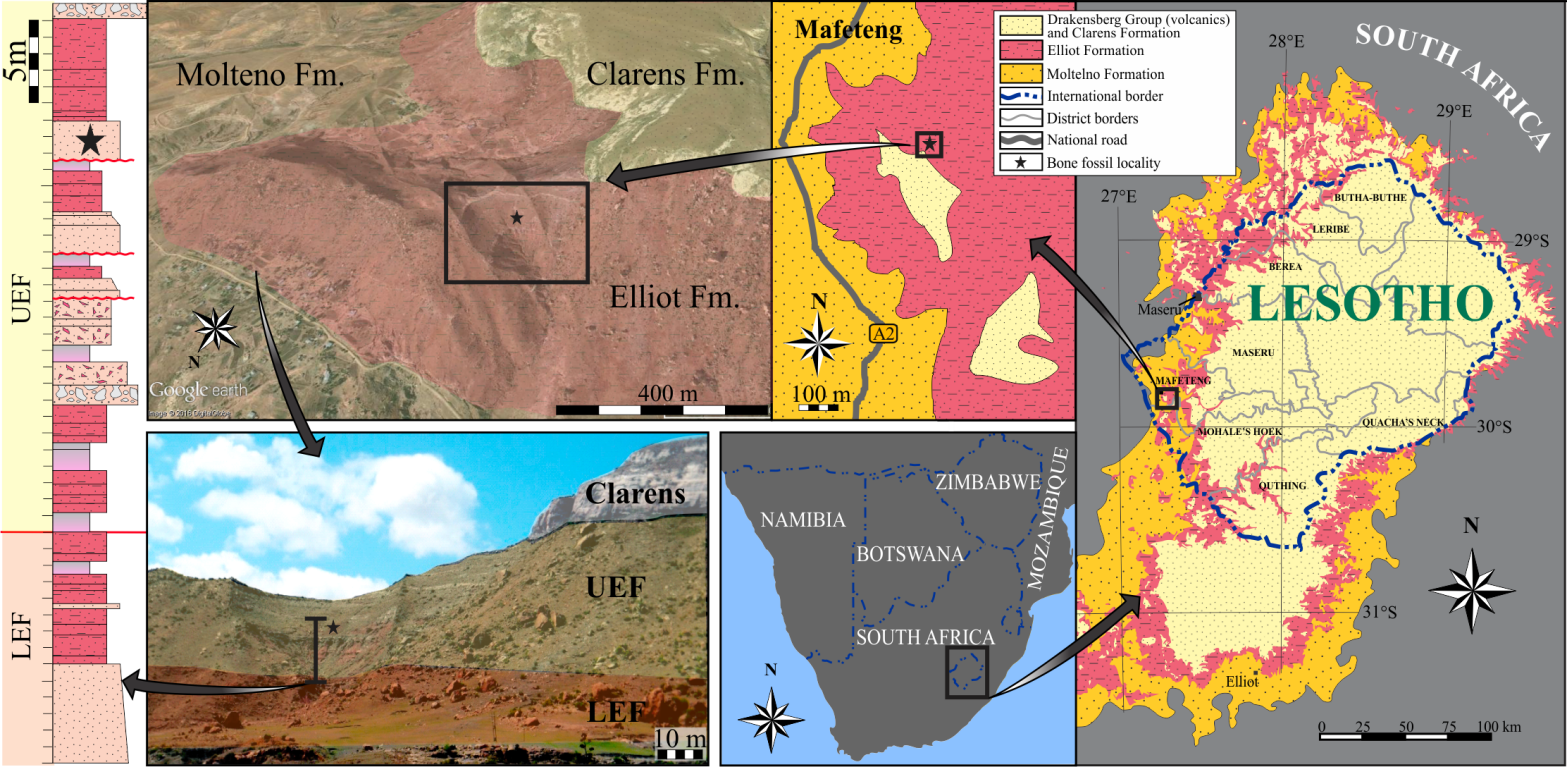
Locality information of the newly discovered *Lesothosaurus diagnosticus* specimen (BP/1/7853) in the upper Elliot Formation (Lower Jurassic) on Likhoele Mountain, Mafeteng District, Lesotho. Anticlockwise succession of black boxes and arrows denote maps of the geographical and geological background on Likhoele, a locality important not only for significant Lower Jurassic fossil discoveries, but also for the relative abundance of *Lesothosaurus diagnosticus* specimens (see inset in Fig. 2). Stars on the maps and sedimentological log indicates the approximate location from where the adult *Lesothosaurus* mandible was extracted: ∼1,876 m above sea level, ∼90 m below the lithostratigraphic contact of the Elliot and Clarens Formations and ∼25 m above the contact between the lower and upper Elliot Formations (LEF and UEF). Google Earth landscape image and photograph have been coloured to indicate lithology. Landscape view, MapData: Google, DigitalGlobe 2016.

**Figure 2:**
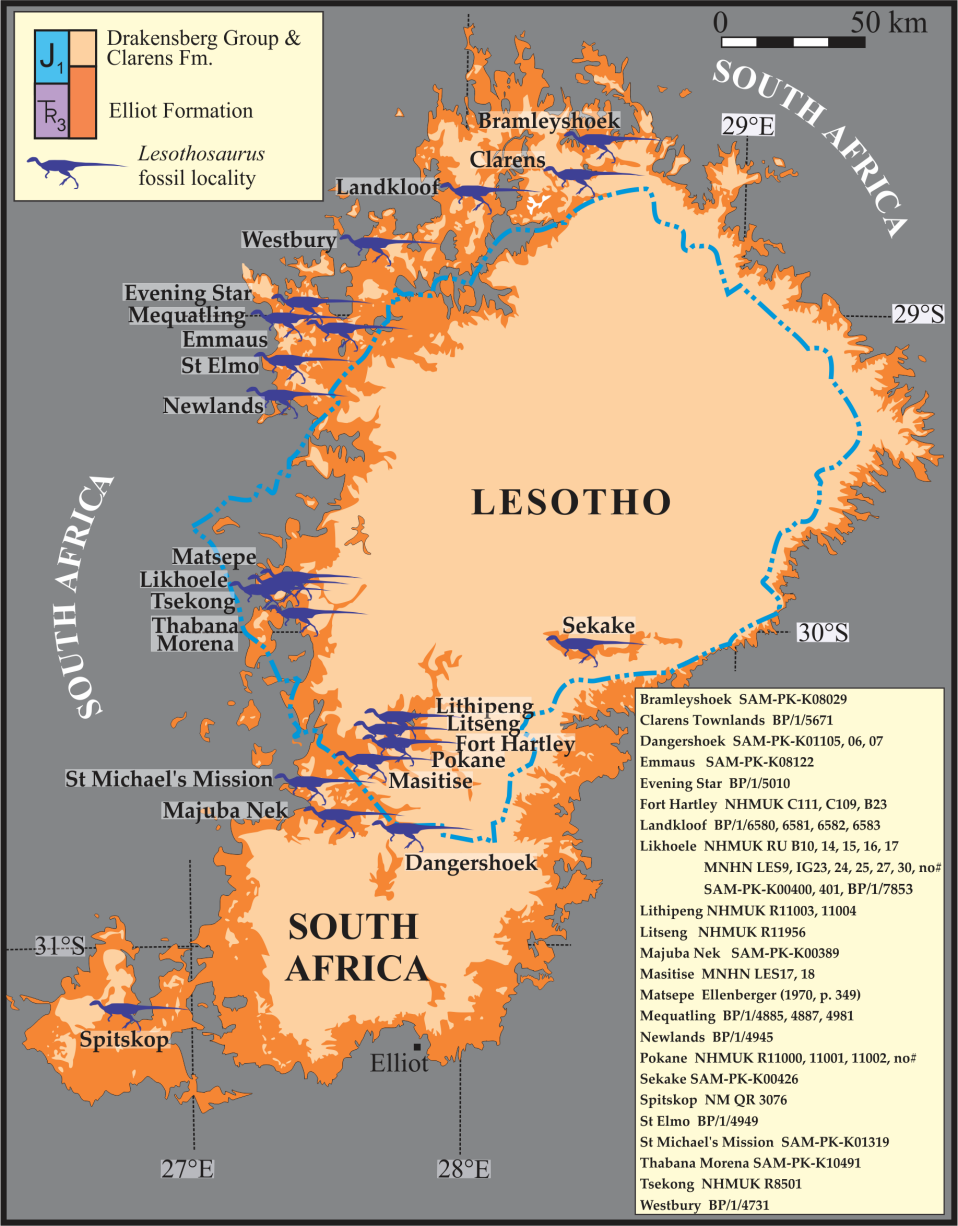
Map of localities for a total of 46 *Lesothosaurus diagnosticus* and *Lesothosaurus* cf. *diagnosticus* specimens in the upper Elliot Formation of Lesotho and South Africa. Most specimens have been assessed first hand. See text for institutional abbreviations and supplement for georeferenced map (Supplemental Information 2). *Lesothosaurus* silhouette adapted from Paul (2010) and used with permission.

The specimen of *Lesothosaurus diagnosticus* (BP/1/7853) described herein has been recently found by the authors within the middle part of the upper Elliot Formation (Lower Jurassic) at Likhoele Mountain (Mafeteng District, Lesotho—Figs. 1 and 2). This is a site renowned for previous fruitful palaeontological fieldwork. The history of the expeditions and collections was meticulously reviewed by Ambrose (1991), and further fieldwork has been carried out by Knoll (2002a) and Knoll (2002d). Some of the important discoveries found at or proximal to our field site at Likhoele Mountain are the original specimens of the first southern African Late Triassic traversodont cynodont, *Scalenodontoides macrodontoides* (Crompton & Ellenberger, 1957), and an early mammaliaform *Erythrotherium parringtoni* (Tsekong village; Crompton, 1964). Furthermore, one of the first described specimens of *Lesothosaurus* (NHMUK RU B17) was also recovered from the northern flank of this mountain (Thulborn, 1970; Thulborn, 1971).

The remains of ornithischian dinosaurs are relatively rare in the upper Elliot Formation or indeed in the upper Stormberg Group as a whole (Knoll, 2005), and only two lineages are represented. In addition to the Heterodontosauridae Kuhn, 1966, three species have been erected: *Fabrosaurus australis* Ginsburg, 1964; *Lesothosaurus diagnosticus* Galton, 1978; and *Stormbergia dangershoeki* Butler, 2005. *Fabrosaurus australis* is generally seen as a *nomen dubium* (Knoll, 2002a; Norman, Witmer & Weishampel, 2004; Butler, 2005). On the basis of a comprehensive review of the evidence at hand, Knoll, Padian & Ricqlès, (2010) made a case for recognising *Stormbergia dangershoeki* as a junior synonym of *Lesothosaurus diagnosticus*. The synonymy was rejected by Maidment & Barrett (2011), but confirmed by Baron, Norman & Barrett (2017). The main difference between the two species is the apparent lack of an obturator process on the ischium in *Lesothosaurus*. However, juveniles often lack well-formed processes for muscle attachment, which grow during ontogeny in response to the mechanical stress induced by a ligament or a tendon (see e.g., Geist & Jones, 1996). Indeed, Guenther (2009: Fig. 11) showed that the ontological appearance of the obturator process of the ischium may be delayed in Neornithischia. Therefore, the evidence currently available strongly suggests that *Lesothosaurus diagnosticus* is the only non-heterodontosaurid known from the upper Elliot Formation.

**Figure 3:**
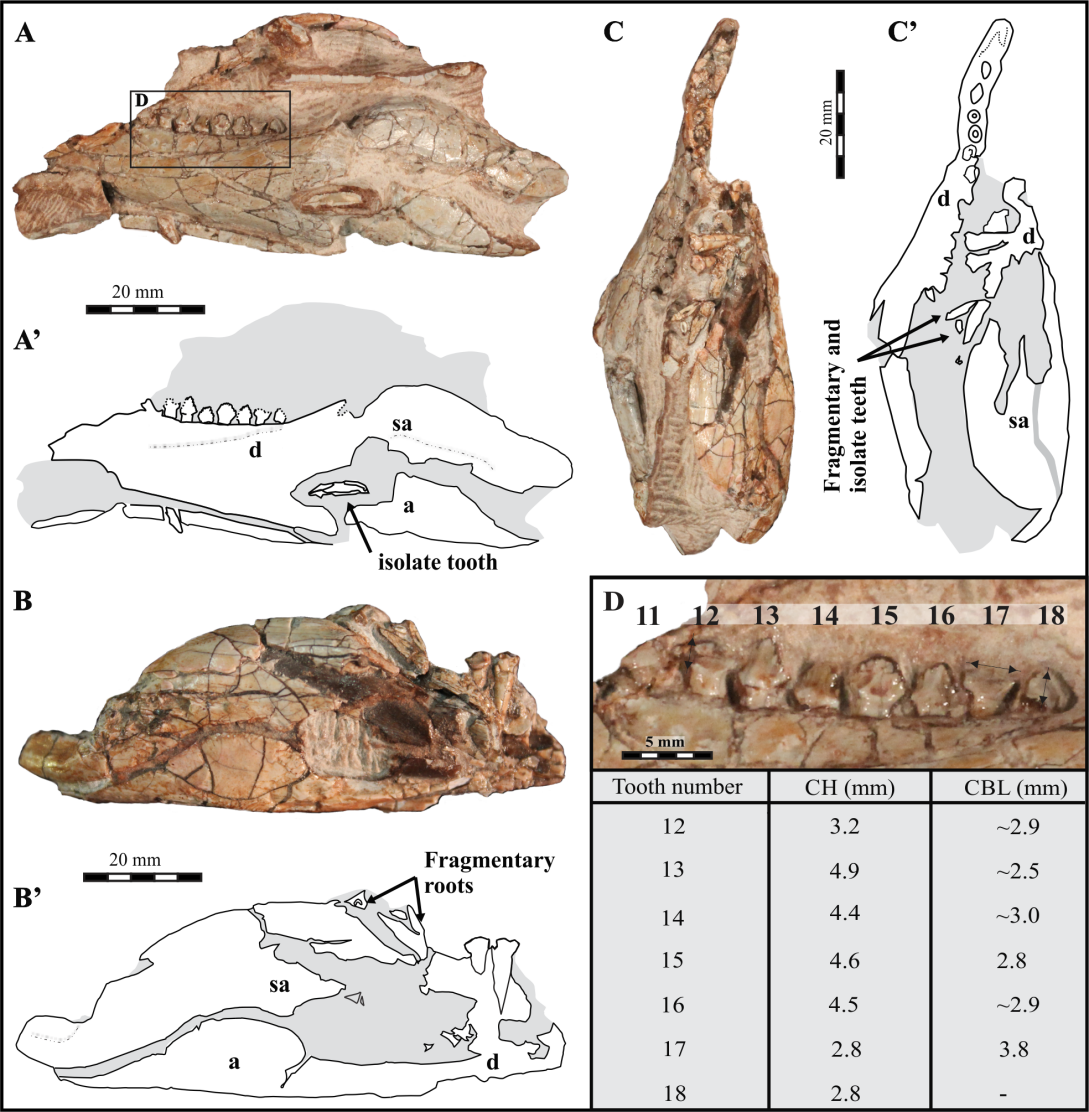
Partial mandible of an adult *Lesothosaurus diagnosticus* (BP/1/7853) from the Lower Jurassic upper Elliot Formation, Likhoele Mountain, Lesotho. (A) Left mandible in medial view with rectangle showing the portion of the dental fragment studied via CT scanning. Black arrow(s) indicate fragmented and loose teeth. (B) Right mandible, cracked and impregnated with Fe-and Mn-oxides making it difficult to scan; showing two functional fragmentary dentary teeth. (C) Specimen in dorsal view. (D) Tentative numbering of teeth exposed in the left dentary achieved by comparison to Sereno (1991: Fig. 13F–13H) and used for the sake of ease of discussion (see Fig. S1). Measurements of the exposed teeth are indicated in the table beneath. The apparent crown height (CH), measured as straight line distance between apex of tooth to neck, and crown basal length (CBL), which was measured as the straight line distance (width) between the mesialmost and distalmost denticles. All measurements were taken using ImageJ software. Abbreviations: a, angular; d, dentary; sa, subangular. Grey denotes the matrix.

Preparation of BP/1/7853 exposed the two rami of the mandible and small denticulate teeth (height from base of crown: 1.5–3 mm—Fig. 3). The specimen is cracked and incomplete rostrally, but it is determined to belong to *Lesothosaurus diagnosticus* on the basis of similarities in dental and mandibular morphology to specimens of this species described by Sereno (1991) and the *Lesothosaurus* sp. specimens described by Knoll (2002a), Knoll (2002b) and Knoll (2002c), which have been subsequently identified as *Lesothosaurus diagnosticus* (Porro, Witmer & Barrett, 2015). The crown morphology and proportions (e.g., denticle density) match those of *Lesothosaurus* specimens NHMUK RU B17, NHMUK R8501, and MNHN LES17. BP/1/7853 is larger (∼95 mm, length of preserved part of left mandible) than any mandible of this taxon found to date and the discovery of larger specimens was not unexpected (see Knoll, Padian & Ricqlès, 2010). Analysis of the mandible of this adult *Lesothosaurus* is important because the results can aid the understanding of the: (1) biology of dental replacement in this particular taxon; (2) feeding strategies of early ornithischians; and (3) niche partitioning in the semi-arid continental settings of Early Jurassic Gondwana. The latter could assist in the explanation as to why *Lesothosaurus* is relatively poorly represented in the fossil record of the upper Stormberg Group in comparison to other sympatric herbivorous dinosaurs and cynodonts in the Early Jurassic within the main Karoo Basin.

### Geological and palaeo-environmental settings

The specimen was found in an amphitheatre-like outcrop (29°51′01.20″S; 27°16′08.45″E) within the Lower Jurassic upper Elliot Formation (Stormberg Group, Karoo Supergroup) at Likhoele Mountain, in the Mafeteng District of Lesotho (Figs. 1 and 2). The Upper Triassic-Lower Jurassic Elliot Formation, together with the unconformably underlying Molteno and conformably overlying Clarens Formations, is part of the Stormberg Group. The Elliot Formation has been lithostratigraphically subdivided into the informal lower Elliot Formation (LEF), and the upper Elliot Formation (UEF) (Bordy, Hancox & Rubidge, 2004a).

*Lesothosaurus* is exclusively found in the upper Elliot Formation in Lesotho and South Africa (Knoll & Battail, 2001; Knoll, 2002a; Fig. 2 and Table S1). Being restricted to these Lower Jurassic continental red beds, it is accepted that this ornithischian dinosaur lived under conditions of increasing aridity with episodic dry intervals of uncertain length and regularity (Bordy, Hancox & Rubidge, 2004a; Bordy, Hancox & Rubidge, 2004b; Bordy, Hancox & Rubidge, 2004c; Sciscio & Bordy, 2016). This palaeoclimatic setting is indicated not only by geochemical data but also by the changing patterns of fluvial sedimentation from perennial rivers (LEF) to flash-flood dominated, ephemeral stream and lake systems (UEF). The latter also contains an abundance of calcareous palaeosols, *in situ* and reworked pedogenic carbonate nodules, desiccation cracks, interbedded wind-blown deposits, etc. In addition to these semi-arid palaeoclimate indicators, the dearth of non-woody and woody plant taxa also supports a relatively dry environmental setting in the Early Jurassic of southern Gondwana. According to Bamford (2004), the palaeobotanical record of the Elliot and overlying Clarens Formations is a low diversity assemblage comprising fossil bennettitales (*Otozamites*), conifers (*Sphenolepidium*), sphenophytes (*Equisetites*) as well as gymnospermous woody taxa (e.g., *Podocarpoxylon, Agathoxylon*).

### Jaw action and dental occlusion

In 1971, Thulborn published a study on the tooth macrowear and jaw action in *Lesothosaurus diagnosticus* (the specimens studied were then referred to as *Fabrosaurus australis*). Compared with those of other Ornithischia, the small cheek teeth of *Lesothosaurus* bear fine denticulations that enabled them to have an efficient cutting action. Thulborn (1971) and Thulborn (1978) suggested that during their abrasion a self-sharpening mechanism of the teeth permitted slicing, and that the macrowear pattern of two wear surfaces on each crown was caused by the interlocking of the upper and lower teeth during occlusion. This is consistent with an orthal jaw action and represents the simplest feeding mechanism proposed for Ornithischia.

Subsequently, most authors endorsed this model (see e.g., Galton, 1978; Galton, 1986; Weishampel, 1984; Norman & Weishampel, 1985; Crompton & Attridge, 1986; Weishampel & Norman, 1989; King, 1996; Peng, 1997). However, the “Fabrosauridae” (including *Lesothosaurus*) do not present attrition facets (i.e., tooth-to-tooth wear surfaces) as for Galton (1978). In other words, there would be no self-sharpening mechanism. Sereno (1991) later stated that this was incorrect and that wear facet did develop in the dentition of *Lesothosaurus* although biplanar facets appeared only locally. For instance, NHMUK PV R8501 (crushed skull and mandible; (Sereno, 1991; Figs. 2 and 3) features several, complete crowns showing no wear. In this specimen, the maxillary and dentary tooth crowns would not have interlocked uniformly when the jaws closed. This view was shared by Norman & Weishampel (1991), who suggested too that tooth wear would be developed sporadically along the tooth row in *Lesothosaurus*. Due to the absence of well-defined and regular wear facets, these authors suggested that little oral food processing is likely to have occurred. In most specimens of *Lesothosaurus* available, there is indeed very little indication of any significant oral processing. The specimen NHMUK PV R11956 (crushed cranial and dentary elements), for example, displays different states of tooth eruption, but no macrowear on any tooth. Nevertheless, among the isolated crowns studied by Thulborn (1971), teeth such as NHMUK RU B17C18 do have both the mesial and distal edges worn, whereas others such as NHMUK RU B17C28 have only one well-worn side. These apparently conflicting observations between *in situ* and isolated teeth were accounted for by assuming frequent tooth replacement (Thulborn, 1971; Thulborn, 1978).

The controversy surrounding the model presented by Thulborn (1971) stemmed in part from the absence of usable description of associated crania and mandibles of *Lesothosaurus.* NHMUKRU B23, in addition to being incomplete and distorted, has very poorly preserved teeth. Specimen MNHN LES 17, is a fairly complete, articulated, and undistorted skull of a juvenile individual of *Lesothosaurus diagnosticus* (Knoll, 2002b) which shows that interlocking did occur in *Lesothosaurus* jaws (Knoll, 2002b: Fig. 1A, 1B; Knoll, 2008). However, the diagram of Thulborn (1971: Fig. 6) is an abstractly perfect case: the interlocking is not uniform along the jaw. Moreover, examination of MNHN LES 17 confirms that the teeth of *Lesothosaurus* are positioned at a roughly oblique angle (imbricated manner) to one another or ‘*en échelon*.’ These observations on the teeth are consistent with an essentially orthal or near-vertical tooth-to-tooth shearing motion between the maxillary and dentary teeth in *Lesothosaurus*. The probable absence of muscular cheeks in *Lesothosaurus* is consistent with a simple oral ‘processing’ (slicing) of the food before swallowing (Knoll, 2008).

The new, large mandible presented here contributes to the knowledge of the jaw movement, tooth wear, and tooth replacement in adult *Lesothosaurus*. Ultimately, it is useful to understand the feeding strategy of this taxon in the semi-arid Karoo ecosystem of the Early Jurassic of Gondwana.

## Materials and Methods

The specimen described here (BP/1/7853) is entrusted to and accessible at the Evolutionary Studies Institute (ESI; previously Bernard Price Institute for Palaeontological Research), University of the Witwatersrand, Johannesburg, South Africa. The specimen was discovered during prospecting under a field permit provided by the Lesotho Government Department of Mines and Geology (permit number: NR/M/E/10).

After some mechanical preparation, using conventional fossil preparation equipment, measurements of exposed teeth in the mandible were performed on photographs of the specimen with ImageJ. For histological analysis, a section of the right mandible was taken (100 µm thick) and processed utilising the methodology outlined in Padian & Lamm (2013).

When still encased in rock, the specimen was scanned using computed tomography (µCT). CT-scanning was carried out at the University of Stellenbosch using a General Electric Phoenix v—tome—x L 240 micro-CT scanner with 25 µm inter-slice spacing (voltage: 150 kV; current: 160 uA). The dataset obtained was subsequently segmented and 3D rendering was performed with VGStudio MAX 2.2 (Volume Graphics GmbH, Heidelberg, Germany) visualisation software. Teeth were individually and manually segmented using the region growing tool and teeth were then extracted and coloured. Video composites of scans are available as electronic supplements to this article. The raw dataset and final digital 3D reconstruction have been deposited in an online repository hosted by the ESI and are available upon request.

## Results

Mechanical preparation revealed that the rostral portion of both dentaries is broken away. The left dentary (Figs. 3A, 3C) is, however, much better preserved than the right one (Figs. 3B, 3C), which was exposed in the outcrop. The preserved caudal portion of the left dentary bears eight teeth with complete or fragmentary crowns and five tooth sockets. The alveoli of the five empty tooth sockets teeth can be seen rostrally in Fig. 3C in the dorsal view of the specimen (they cannot be seen in lateral view in Fig. 3A). The right mandible shows only two exposed teeth within the ramus and two isolated teeth associated with the ramus being fragmentary (Fig. 3B). There are four isolated teeth altogether associated with this specimen.

In general the dentary of BP/1/7853 is gently arched. Its dorsal and ventral margins taper slightly rostrally (Figs. 3A, 3C). The coronoid eminence is low. The margin of the external mandibular fenestra of the left mandible has been cracked by the dissociation of the angular and surangular along the former sutural contact. The external mandibular fenestra of the right mandible is better preserved and shows a large and roughly oval outline. The retro-articular process, which is well-preserved on the right fragment, projects strongly caudally. As preserved, the maximum total length of the left mandible in lateral view is 95 mm. Measurements of the exposed teeth in the left dentary are presented in Fig. 3D.

### Bone histology

**Figure 4:**
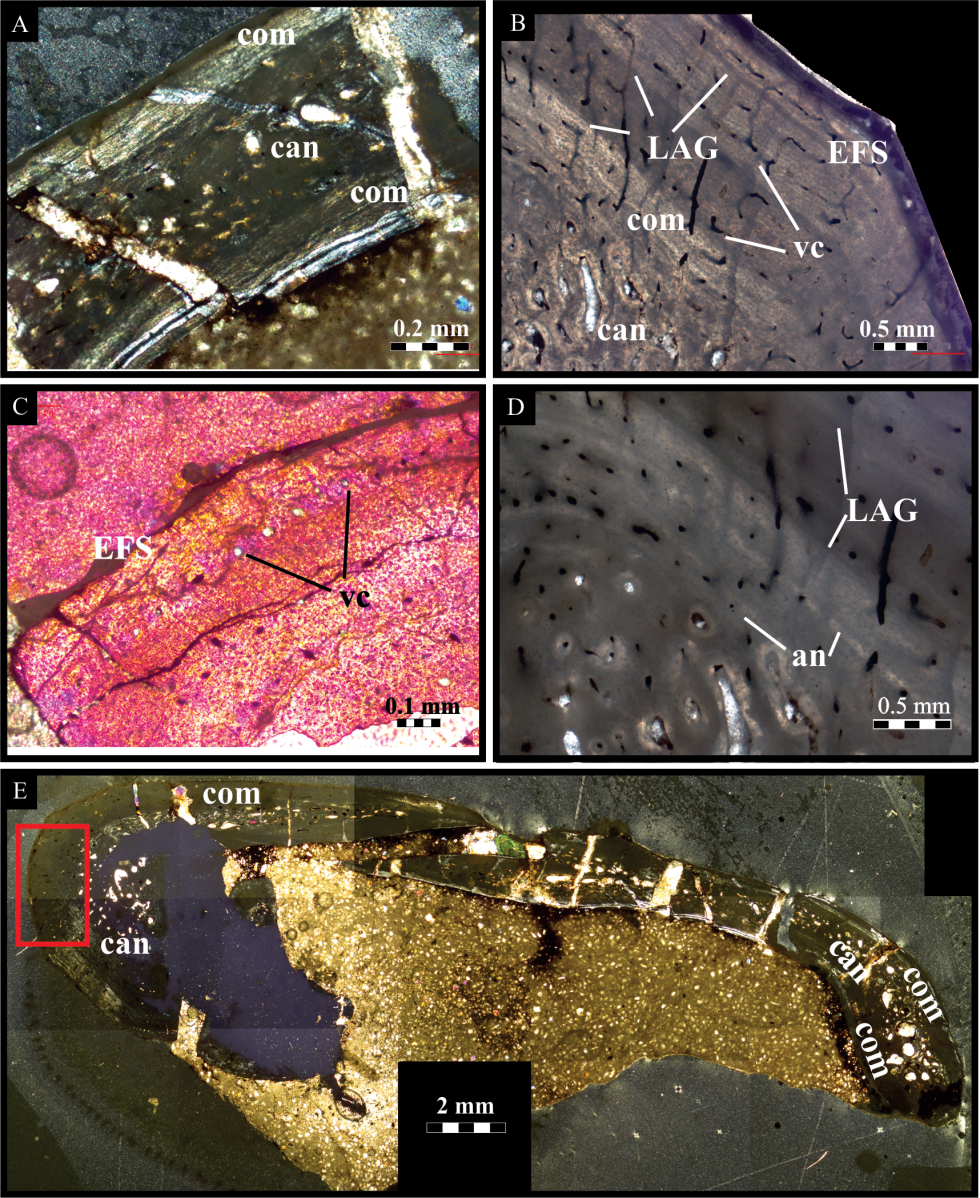
Bone histology (cross-sectional views) of BP/1/7853. (A) Primary bone is well-preserved in the compact bone, while some remodelling occurs in the cancellous bone. (B). Primary bone shows longitudinal and reticular vascular canals and three lines of arrested growth. (C) Vascular canals decrease in number close to the bone surface. (D) Two annuli and three lines of arrested growth were deposited in the primary bone. (E) Radial canals occur in one area, indicated by a red rectangle. Abbreviations: an, annulus; can, cancellous bone; com, compact bone; EFS, external fundamental system; LAG, line of arrested growth; vc, vascular canal.

The primary bone is mostly well-preserved in BP/1/7853. Secondary remodelling, indicated by large secondary osteons, is restricted to the cancellous bone of the central area (Fig. 4A). The compact bone consists of parallel-fibered bone that is vascularised by reticular and longitudinally vascular canals and primary osteons (Fig. 4B). They decrease in number closer to the bone surface (Fig. 4C), indicating that growth slowed down considerably. Two annuli (Fig. 4D) and three lines of arrested growth (LAGs) were deposited in the primary bone (Fig. 4B), followed by an external fundamental system (EFS) at the bone surface (Fig. 4B), which may contain four LAGs. In one area, indicated in Fig. 4E, radial vascular canals occur, probably due to mechanical stress. The development of an EFS shows that growth has ceased as the animal reached somatic maturity, possibly after a minimum of five years as indicated by the deposition of two annuli and three LAGs in the primary bone. Considering the presence seven LAGS (of which four are EFS), the individual can be tentatively estimated to have been a minimum age of nine years old at death.

### Left mandible

**Figure 5:**
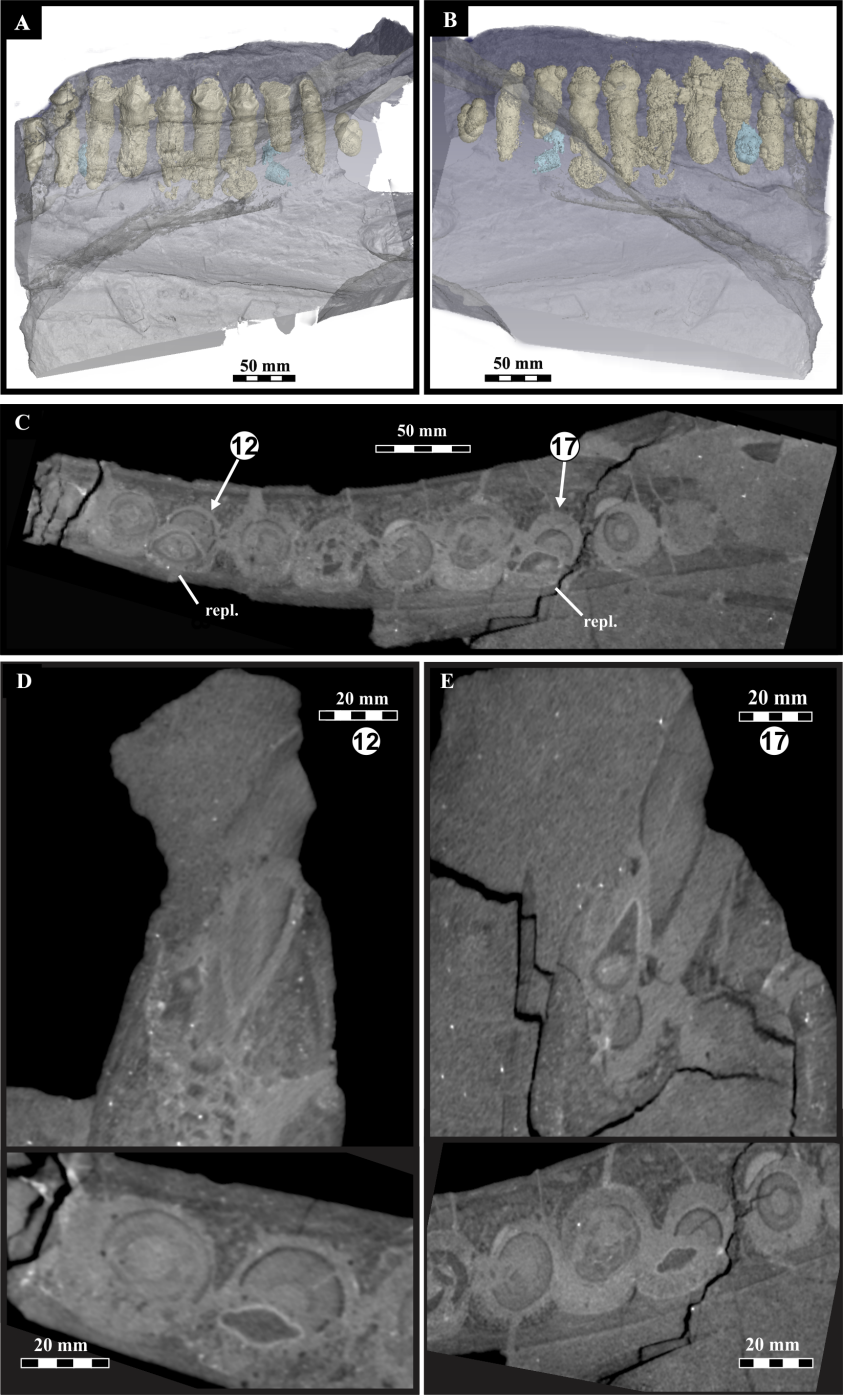
*Lesothosaurus diagnosticus*, BP/1/7853, left mandible microCT scan data showing dentition. (A) Buccal and (B) lingual views of 3D image rendering of the left dentary showing functional (in cream) and replacement (in blue) teeth; (C) horizontal section of tooth row with indication of assumed tooth positions 12 and 17 and the corresponding replacement teeth; (D) coronal and horizontal sections of tooth position 12 and its respective replacement tooth; (E) coronal and horizontal sections of tooth position 17 and its replacement tooth. (See also Videos S1– S3). Abbreviation: repl., replacement tooth.

Crown positions 11–18 (Figs. 5A–5C; see Fig. S1) have been estimated based on Sereno’s (1991: Figs. 13F–13H) reconstruction of the mandible in *Lesothosaurus diagnosticus*. We believe that the ninth tooth socket of the scanned portion of the specimen was the 18th in life (Fig. 3D). We are aware that such estimation is very tentative, but we assume it to be correct below for the sake of ease of discussion. There is no significant heterodonty: all the teeth are of a relatively uniform size and shape (Figs. 3D, 5A). The enamel of exposed crowns is smooth. In transverse cross-section (Figs. 5C– 5E), the crowns are symmetrically oval to diamond shaped. The crowns are triangular, labiolingually and mesiodistally expanded immediately dorsal to the neck, and show fine, short, and rounded denticles (∼9 per tooth: ∼4 per mesial and distal sides and one apical) that are angled upwards at <45 degrees from the tooth long axis (Figs. 3D, 5B). The apical denticle is slightly wider mesiodistally than the flanking denticles, with all denticles varying in size along the mesial and distal edges of the crown. Those denticles directly adjacent to the apical denticle are usually smaller (typified by tooth 16; Fig. 3D) than those closer to the base of the crown. The median position of the apical denticle of each tooth results in a relatively symmetrical shape in labiolingual view (Figs. 3 and 5). The crowns are closely packed (Figs. 3D, 5A–5C) and arranged ‘en échelon’.

In BP/1/7853, the X-ray attenuation contrast of teeth is sufficiently different from that of the surrounding bone and matrix to allow for comprehensive rendering of several generations of teeth (both erupted and non-erupted) within the mandible (Fig. 5). The teeth placed more rostrally (outside rectangle in Fig. 3A) are not considered here.

Systematic tooth-to-tooth wear facets are absent from the teeth of the left dentary with exception of teeth at positions 12, 17 and 18. These are apically truncated and show only the basalmost denticle of the crown for functional teeth 12 and 17 (Figs. 3D, 5D, 5E). The truncated surfaces are rounded. Based on the extent of the wear facets, approximately equal degrees of wear appear on functional teeth 12 and 17 while 18 is more worn. Active replacement is only present at tooth positions 12 and 17 (Figs. 5D, 5E) in the scanned fragment. The replacement teeth are seen on the lingual side of the functional tooth and slightly mesial to it (Fig. 5). Both replacements appear to be exploiting the root of the functional tooth (Figs. 5D, 5E). The replacement tooth at position 12 appears to be better developed and lies closer to the neck of the functional tooth than that at position 17. The roots of all teeth are elongate and taper distally, with those of the rostralmost teeth being the longest. For possible taphonomic reasons the root of functional tooth 16 is broken and the replacement tooth, ventral to tooth 17 is also broken at the neck (Figs. 5B, 5E). Functional tooth crown 14 is inserted more ventrally within the dentary than the other exposed crowns. This is visible in photographs (Fig. 3D) and in the CT scan images (Figs. 5A, 5B) where the distance between the crown apex and the dorsal margin of the dentary (0.4 mm) is less than in the other teeth (av. 0.9 mm).

## Discussion

Although detailed descriptions of *Lesothosaurus* skull elements do exist (Thulborn, 1970; Galton, 1978; Gow, 1981; Sereno, 1991; Knoll, 2002b; Knoll, 2002c; Porro, Witmer & Barrett, 2015; Barrett et al., 2016), neither juvenile nor adult *Lesothosaurus* mandibles have been studied internally in much detail to date. Maxillary lengths for larger *Lesothosaurus* specimens range from ∼52 mm (BP/1/6581; Barrett et al., 2016) to ∼70 mm (fragmentary maxilla of NM QR3076; Fig. S2). The maxilla of NM QR3076, (see Fig. S2), the largest *Lesothosaurus* specimen known to date, suggests it is from an individual of a size comparable to that of BP/1/7853. Histological analyses of NM QR3076 (from mid-shaft of femur) suggested it reached somatic maturity in approximately four years (Knoll, Padian & Ricqlès, 2010). This is consistent with histological analyses of BP/1/7853, in which adult age was attained after approximately five years. Bone histology from the large adult individual studied herein confirms that smaller sized individuals of *Lesothosaurus* are likely to be immature.

The study of tooth replacement pattern in reptiles was pioneered by Edmund (1960) and Edmund (1962) who conceptualised dental replacement ‘waves’ (Zahnreihen) in a back to front direction along the jaw with alternate teeth undergoing replacement. Although originally widely accepted, further studies have shown that tooth replacement patterns are more complicated and that replacement waves can reverse at various places along the jaw (Whitlock & Richman, 2013). In ‘fabrosaurids’ (*Lesothosaurus*), Thulborn (1978) initially suggested that rapid tooth replacement occurred in waves with alternating young and old teeth, as in standard reptilian patterns (Edmund, 1960), and that teeth had short duration of functional use despite wear. This was later opposed by Hopson (1980).

From the specimen BP/1/7853, computed tomography revealed two replacement teeth on the lingual side of the worn teeth at positions 12 and 17 (Fig. 5). It appears that the new buds were growing into and exploiting the central pulp cavity of the overlying teeth as seen in Fig. 5D and 5E. Wear on functional teeth at these positions (Figs. 5A–5C), as well as on tooth position 18 (not undergoing replacement), is extreme with missing tips and fractured margins appearing rounded and smooth due to wear. Tooth wear in the other teeth of the left dentary is negligible (Fig. 3D).

The two replacement teeth visible in the scans (Figs. 5D, 5E) are erupting asynchronously judging from their different depth with respect to the alveolar margin of the dentary. This would allow for replacement at tooth position 12 to be sooner than that at position 17. This also holds true for other functional teeth in the dentary, for example, tooth 14 sits lower in the dentary than the other teeth and may have erupted later. We have inferred different timing of eruption as reflected in crown height within the dentary. This can be noted in other specimens, e.g., SAM PK K00426, assigned to *Lesothosaurus* and reported by Sereno (1991: Fig. 4, p. 178), which shows the right maxillary dentition in which replacement is advanced at the most worn tooth position (6), and differentiation in timing of eruption is also noted (i.e., tooth positions 4, 9). There is a generally noted varied degree of emergence of the teeth exhibiting less wear and not undergoing immediate and visible replacement The lack of teeth undergoing replacement (i.e., here only two out of eight scanned teeth, or for SAM PK K00426, approximately one of thirteen) likely suggests slow replacement rates. This would be contra Thulborn (1971) and Thulborn (1978) who suggested rapid tooth replacement due to wear and root resorption. Root resorption is not visible in BP/1/7853. However, the root of the tooth at position 17 appears shorter in comparison to the situation in the other functional teeth. We infer that tooth replacement was an asynchronous, continuous and slow process in mature *Lesothosaurus*.

On the basis of BP/1/7853 and other, including more complete, specimens (such as MNHN LES 17), we assume orthal jaw motion in *Lesothosaurus diagnosticus*, as previously suggested (Thulborn, 1971; Weishampel, 1984; Williams, 2010). The microwear study by Williams (2010) supports near vertical shearing motion of the jaw and provides evidence against any propalinal jaw action in *Lesothosaurus*. Imprecise occlusion is supported by the macrowear and microwear features on the maxillary and dentary teeth (specimen NHMUK R11956). The evidence from BP/1/7853 in conjunction with that provided by other specimens (Sereno, 1991; Knoll, 2002b; Knoll, 2008; Barrett et al., 2016) shows that tooth wear is not conspicuous in most *Lesothosaurus* specimens. Post-depositional/taphonomic processes likely account for the fractured nature of the roots seen in the CT scans (Figs. 5A, 5B). The absence of wear facets on the teeth means that consistent, abrasive tooth-to-tooth wear was not as great during chewing and is restricted to three teeth.

Hypothetically, the combination of the orthal movement, imprecise interlocking, asynchronous eruption, and the ‘*en échelon’* tooth arrangement could result in increased force acting on the coronal margin of the tooth while chewing. This may have impacted on the way teeth at positions 12, 17, and 18 are worn and truncated (i.e., coronal tips missing—Figs. 3D, 3E, 5A, 5B), and becomes an important consideration if replacement of teeth was not *en bloc* but rather sporadic, i.e., causing some teeth to emerge higher/lower from the jaw margin. For example, in BP/1/7853, the tooth at position 14 (Fig. 3D), potentially, represents a recent replacement which had not yet reached its eventual position in the dentary row as it sits lower than the neighbouring teeth and shows no wear. During jaw closure, teeth protruding higher than the adjacent teeth (in this case, tooth positions 13, 15 relative to 14) would be placed under greater stress leading to micro-fractures and accounting for the (later) fractured apex.

*Lesothosaurus diagnosticus* contemporaneously occurs with several heterodontosaurid ornithischians in the Elliot Formation. In comparison to the heterodontosaurids, *Lesothosaurus* is considered to represent one of the earliest ornithischians in the Elliot Formation, and as such does exhibit more simplified dental (and cranial) morphology in comparison to the former. Teeth in this taxon are ‘leaf-shaped’ with a neck separating crown and root which is not seen in the heterodontosaurids (Norman et al., 2011; Porro, Witmer & Barrett, 2015). In particular, it is noted that the degree of dental wear is significantly different between the two, with *Lesothosaurus* specimens often displaying limited but consistent wear that is typified by missing tips (Thulborn, 1971; Weishampel, 1984; Williams, 2010). Heterodontosaurids, conversely, have heterodont dentition which shows strong wear that changes not only in its degree but also in the angulation along the dental battery (Norman et al., 2011; Sereno, 2012). In association with the more complex dental specialisation, complex jaw movements have been proposed for heterodontosaurids due to the tooth wear and sutural relationships of the bones in the lower jaw, the latter being specifically applied to *Heterodontosaurus tucki* (Crompton & Attridge, 1986; Porro et al., 2011; Norman et al., 2011; Sereno, 2012). Likewise, a cheek recess is also more pronounced in heterodontosaurids in comparison to *Lesothosaurus* although not all heterodontosaurids show this conditions equally, i.e., *Abrictosaurus* (Norman et al., 2011). Because of the differences in cranial morphology and dental specialisation and wear, it has been proposed that tooth replacement rates in heterodontosaurids are similar to *Lesothosaurus* (Porro, Witmer & Barrett, 2015). Norman et al. (2011) note that the low number of alveolar foramina on the medial surface of the maxilla, specifically in the well-studied genera *Heterodontosaurus* and *Fruitadens*, supports slow, sporadic tooth replacement rates. Different genera of heterodontosaurids show variability in the characteristics of jaw action, tooth replacement rate, and wear (Butler, Porro & Norman, 2008; Norman et al., 2011; Porro et al., 2011; Sereno, 2012), and this is likely a function of their stratigraphic placement in the Elliot Formation in addition to suggested niche partitioning (Porro et al., 2011).

The semi-arid climatic regime represented by sediments of the upper Elliot Formation suggests highly seasonal and sporadic rainfall. This would have influenced plant type and availability which in turn would have had bearing on dietary choices and tooth wear in adult *Lesothosaurus* and the heterodontosaurids. Fossil plant and palaeopalynological studies from the Elliot Formation suggest low diversity assemblages (Bamford, 2004; Barbolini, 2014); however, limited diversity in fossil plant assemblages is not an accurate reflection of the floral biodiversity at the time but rather of the complex and limiting taphonomic filters typical of semi-arid settings and their low preservation potential for plant material (cf. Pšenička & Opluštil, 2013; Gastaldo & Demko, 2011).

It is difficult to say what plant communities dominated the semi-arid environment of the upper Elliot Formation, or how they have affected the animals living in this environment. In assuming low diversity of plants available for consumption, especially during the dry seasons, restricted dietary choices would have resulted for plant eaters This would lead to an increased consumption of tougher to ingest, drought-tolerant plants and xerophilous vegetation bearing reduced or abrasive silicate-rich leaves and likely increasing differential stress on teeth in *Lesothosaurus* caused by asynchronous replacement. Variability of tooth wear is also likely linked to the seasonal variability within the floral community which is closely tied to environmental pressures (e.g.,  water stress). Lastly, combining poor grazing with dietary diversification, such as omnivory, could ultimately influence tooth wear patterns. It is noted that the rate and pattern of tooth wear in animals with flexible dietary requirements may be more variable (Lister, 2014). Contrastingly, traditionally it is considered that habitat and not explicitly diet may play a role in the severity of wear (microwear specifically; Xia et al., 2015).

Norman & Weishampel (1991) proposed that *Lesothosaurus* probably only consumed highly nutritious, soft-bodied fructifications and shoots. Barrett (2000), in contrast, favoured the theory of an omnivorous diet in *Lesothosaurus*. Diet speculation from co-occurring heterodontosaurid ornithischians suggest that some, such as *Abrictosaurus*, with its steeply wore teeth and orthal jaw action, may be comparable with *Lesothosaurus* in eating less fibrous vegetation or being intermittently omnivorous (Porro et al., 2011). However, *Lesothosaurus* specimens’ show a lack of significant wear on most teeth and may indicate that the food consumed was not considerably abrasive, and that wear is a function of differential stresses on teeth caused by slower asynchronous replacement. Only three teeth in BP/1/7853 show some degree of wear, while the rest display no wear at all and two of the most worn teeth were underlain by replacement teeth. This suggests that the teeth were mainly replaced while the crown was not yet completely worn. This is also seen in other specimens, such as SAM PK K00426 in which the most worn teeth appear also to be those undergoing replacement. The case shown by BP/1/7853 tooth 18, which is nearly completely worn and is not undergoing replacement, is not the most common condition for *Lesothosaurus*. Additionally less worn functional teeth that are not undergoing replacement are not equally high and suggests asynchronous replacement.

Feeding mechanisms and variability in tooth wear may also have a bearing on the function of the beak in *Lesothosaurus*. Sereno (1991), Norman, Witmer & Weishampel, (2004), Knoll (2008) and more recently Porro, Witmer & Barrett (2015) have discussed and provided evidence for a keratinous beak occurring at the rostral end of the jaw as evidenced by the rough, rugose texture of the bone in this area. Norman & Weishampel (1991) proposed *Lesothosaurus* used the beak for feeding on selectively soft fruits and shoots. The feeding functionality alluded to in Norman, Witmer & Weishampel, (2004) may have bearing on the lack of tooth wear, if the beak was used to feed on seed stores left by annual plant communities. Thus, seed cracking utilising the beak would be in line with reduced oral processing, and would be important in the dry season where seeds can serve as highly nutritious repositories. However, this is contra the shape and functionality otherwise assigned to the teeth of *Lesothosaurus*.

## Conclusion

The adult specimen of *Lesothosaurus diagnosticus* examined in the present study suggests that the teeth were replaced asynchronously, rather than *en bloc*, and apparently after little wear. This staggered tooth replacement and modest amount of wear is also apparent in other specimens of *Lesothosaurus* from younger individuals. If the diet of *Lesothosaurus* was heavily reliant on fructifications and/or young shoots (Norman & Weishampel, 1991), these animals would have been exposed to extreme food limitation during most of the year, given the semi-arid setting. *Lesothosaurus* was more probably adapted to eating what was readily available or in abundance in its immediate environment at any given time. The facultative omnivory advocated by Barrett (2000) would have allowed *Lesothosaurus* to better face episodes of environmental stress (i.e., prolonged, irregular droughts), which provoked fluctuations in the amount and type of food available.

##  Supplemental Information

10.7717/peerj.3054/supp-1 Figure S1 The nine preserved dentary alveoli from tooth positions 10 –18 of BP/1/7853 in comparison to the adapted illustration of Sereno’s (1991: Fig. 13H) The nine preserved dentary alveoli from tooth positions 10 –18 of BP/1/7853 in comparison to the adapted illustration of Sereno’s (1991: Fig. 13H) reconstruction of the mandible in Lesothosaurus. The ninth tooth socket of BP/1/7853 in the scanned fragment was the 18th in life. This is an estimation used for the sake of ease of discussion. Abbreviation: repl., replacement crown.

10.7717/peerj.3054/supp-2 Figure S2 Left maxilla of NM QR 3076 in (A) medial, (B) lateral, and (C) ventromedial view

10.7717/peerj.3054/supp-3 Supplemental Information 1 Locality information pertaining to the 46 *Lesothosaurus diagnosticus* and *Lesothosaurus* cf. *diagnosticus* specimens in the upper Elliot Formatio NHMUK, The Natural History Museum, London
BMNH, British Museum of Natural History, London
MNHM, Muséum National d’Histoire Naturelle, Paris
BPI, Bernard Price Institute of Palaeontology, Johannesburg, South Africa (now ESI –Evolutionary Studies Institute).
NM, National Museum, Bloemfontein
SAM, South African Museum (now Iziko Museum), Cape Town

10.7717/peerj.3054/supp-4 Supplemental Information 2 Georeferences of fossil localities for a total of 46 *Lesothosaurus diagnosticus* and other* Lesothosaurus* cf. *diagnosticus* specimens in the upper Elliot Formation of Lesotho and South Africa Abbreviations
NHMUK, The Natural History Museum, London
BMNH, British Museum of Natural History, London
MNHM, Muséum National d’Histoire Naturelle, Paris
BPI, Bernard Price Institute of Palaeontology, Johannesburg, South Africa (now ESI –Evolutionary Studies Institute).
NM, National Museum, Bloemfontein
SAM, South African Museum (now Iziko Museum), Cape Town

10.7717/peerj.3054/supp-5 Video S1 MicroCT scan images of the left dentary of *Lesothosaurus* BP/I/7853 showing transverse view of replacement tooth behind tooth position 12

10.7717/peerj.3054/supp-6 Video S2 MicroCT scan images of the left dentary of *Lesothosaurus* BP/I/7853 showing transverse view of replacement tooth

10.7717/peerj.3054/supp-7 Video S3 3D image reconstruction and segmentation of teeth in left dentary of *Lesothosaurus* BP/I/7853 showing buccal and lingual view of replacement teeth (shown in blue) behind and underlying teeth at positions 3 and 8

## Abbreviations

BP: Bernard Price Institute of Palaeontology, Johannesburg, South Africa (now ESI —Evolutionary Studies Institute).
NHMUK: The Natural History Museum, London
NM: National Museum, Bloemfontein
MNHM: Muséum National d’Histoire Naturelle, Paris
SAM: South African Museum (now Iziko Museum), Cape Town

## Additional Information and Declarations

### Field Study Permissions

The following information was supplied relating to field study approvals (i.e., approving body and any reference numbers): Field permit was provided and approved by the Lesotho Government Department of Mines and Geology (permit number: NR/M/E/10).

### Data Availability

The following information was supplied regarding data availability: Specimen accession Number: BP/I/7853 (University of the Witwatersrand). Raw data is included as Supplemental Files and raw CT data can be obtained from the Evolutionary Science Institute, University of the Witwatersrand.

## References

[ref-1] Ambrose D (1991). A tentative history of Lesotho palaeontology.

[ref-2] Bamford MK (2004). Diversity of the woody vegetation of gondwanan Southern Africa. Gondwana Research.

[ref-3] Barbolini N (2014). Palynostratigraphy of the South African karoo supergroup and correlations with coeval gondwanan succession. PhD Thesis.

[ref-4] Baron MG, Norman DB, Barrett MP (2017). Postcranial anatomy of *Lesothosaurus diagnosticus* (Dinosauria: Ornithischia) from the Lower Jurassic of southern Africa: implications for basal ornithischian taxonomy and systematics. Zoological Journal of the Linnean Society.

[ref-5] Barrett PM, Sues H-D (2000). Prosauropod dinosaurs and iguanas: speculations on the diets of extinct reptiles. Evolution of herbivory in terrestrial vertebrates: perspectives from the fossil record.

[ref-6] Barrett PM, Butler RJ, Yates AM, Baron MG, Choiniere NJ (2016). New specimens of the basal ornithischian dinosaur Lesothosaurus diagnosticus Galton, 1978 from the Early Jurassic of South Africa. Palaeontologia Africana.

[ref-7] Bordy EM, Hancox PJ, Rubidge BS (2004a). Basin development during the deposition of the Elliot Formation (Late Triassic - Early Jurassic), Karoo Supergroup, South Africa. South African Journal of Geology.

[ref-8] Bordy EM, Hancox PJ, Rubidge BS (2004b). Fluvial style variations in the Late Triassic - Early Jurassic Elliot Formation, main Karoo Basin, South Africa. Journal of African Earth Sciences.

[ref-9] Bordy EM, Hancox PJ, Rubidge BS (2004c). A description of the sedimentology and palaeontology of the Upper Triassic—Lower Jurassic Elliot Formation in Lesotho. Palaeontologia Africana.

[ref-10] Butler RJ (2005). The ‘fabrosaurid’ ornithischian dinosaurs of the Upper Elliot Formation (Lower Jurassic) of South Africa. Zoological Journal of the Linnean Society.

[ref-11] Butler RJ, Porro LB, Norman DB (2008). A juvenile skull of the primitive ornithischian dinosaur Heterodontosaurus tucki from the ‘Stormberg’ of southern Africa. Journal of Vertebrate Paleontology.

[ref-12] Crompton A, Ellenberger F (1957). On a new cynodont from the Molteno Beds and the origin of the tritylodontids. Annals of the South African Museum.

[ref-13] Crompton AW (1964). A preliminary description of a new mammal from the Upper Triassic of South Africa. Proceedings of the Zoological Society of London.

[ref-14] Crompton AW, Attridge J, Padian K (1986). Masticatory apparatus of the larger herbivores during Late Triassic and Early Jurassic times. The beginning of the age of dinosaurs.

[ref-15] Edmund AG (1960). Tooth replacement phenomena in the lower vertebrates. Contribution (Life Sciences Division. Royal Ontario Museum).

[ref-16] Edmund AG (1962). Sequence and rate of tooth replacement in the Crocodilia. Contribution (Life Sciences Division. Royal Ontario Museum).

[ref-17] Galton PM (1978). Fabrosauridae, the basal family of ornithischian dinosaurs (Reptilia: Ornithopoda). Palaontologische Zeitschrift.

[ref-18] Galton PM, Padian K (1986). Herbivorous adaptations of late Triassic and early Jurassic dinosaurs. The beginning of the age of dinosaurs: faunal change across the triassic-jurassic boundary.

[ref-19] Gastaldo RA, Demko TM, Allison PA, Bottjer DJ (2011). The relationship between continental landscape evolution and the plant-fossil record: long term hydrologic controls on preservation. Taphonomy: process and bias through time, topics in geobiology 32.

[ref-20] Geist NR, Jones TD (1996). Juvenile skeletal structure and the reproductive habits of dinosaurs. Science.

[ref-21] Ginsburg L (1964). Decouverte d’un Scelidosaurien (Dinosaure ornithischien) dans le Trias superieur du Basutoland. (Discovery of a Scelidosaurian (ornithischian dinosaur) in the Upper Triassic of Basutoland.). Comptes Rendus Hebdomadaires des Seances de l ’Academie des Sciences (FR).

[ref-22] Gow CE (1981). Taxonomy of the Fabrosauridae (Reptilia, Ornithischia) and the Lesothosaurus myth. South African Journal of Science.

[ref-23] Guenther MF (2009). Influence of sequence heterochrony on hadrosaurid dinosaur postcranial development. The Anatomical Record.

[ref-24] Hopson JA (1980). Tooth function and replacement in the Stormberg Series —implications for aestivation. Lethaia.

[ref-25] King GM (1996). Reptiles and herbivory.

[ref-26] Knoll F (2002a). Les fabrosauridae Galton, 1972. (Dinosauria: Ornithischia): répartition géographique et stratigraphique; systématique et phylogénie.

[ref-27] Knoll F (2002b). Nearly complete skull of *Lesothosaurus* (Dinosauria: Ornithischia) from the Upper Elliot Formation (Lower Jurassic: Hettangian) of Lesotho. Journal of Vertebrate Paleontology.

[ref-28] Knoll F (2002c). New skull of *Lesothosaurus* (Dinosauria: Ornithischia) from the Upper Elliot Formation (Lower Jurassic) of southern Africa. Geobios.

[ref-29] Knoll F (2002d). New field works in the Upper Triassic-Lower Jurassic of Lesotho: preliminary results. Journal of Vertebrate Paleontology.

[ref-30] Knoll F (2005). The tetrapod fauna of the Upper Elliot and Clarens formations in the main Karoo Basin (South Africa and Lesotho). Bulletin de la Societie Geologiques de France.

[ref-31] Knoll F (2008). Buccal soft anatomy in *Lesothosaurus* (Dinosauria: Ornithischia). Neues Jahrbuch für Geologie und Paläontologie, Abhandlungen.

[ref-32] Knoll F, Battail B (2001). New ornithischian remains from the Upper Elliot Formation (Lower Jurassic) of Lesotho and stratigraphical distribution of southern African fabrosaurids. Geobios.

[ref-33] Knoll F, Padian K, Ricqlès AD (2010). Ontogenetic change and adult body size of the early ornithischian dinosaur Lesothosaurus diagnosticus: implications for basal ornithischian taxonomy. Gondwana Research.

[ref-34] Kuhn O (1966). Die reptilien, system und stammesgeschichte.

[ref-35] Lister AM (2014). Behavioural leads in evolution: evidence from the fossil record. Biological Journal of the Linnean Society.

[ref-36] Maidment SC, Barrett PM (2011). The locomotor musculature of basal ornithischian dinosaurs. Journal of Vertebrate Paleontology.

[ref-37] Norman DB, Crompton AW, Butler RJ, Porro LB, Charig AJ (2011). The Lower Jurassic ornithischian dinosaur Heterodontosaurus tucki Crompton and Charig, 1962: cranial anatomy, functional morphology, taxonomy and relationships. Zoological Journal of the Linnean Society.

[ref-38] Norman DB, Weishampel DB (1985). Ornithopod feeding mechanisms: their bearing on the evolution of herbivory. The American Naturalist.

[ref-39] Norman DB, Weishampel DB, Rayner MV, Wootton RJ (1991). Feeding mechanisms in some small herbivorous dinosaurs: processes and patterns. Biomechanics in evolution.

[ref-40] Norman DB, Witmer LM, Weishampel BD, Weishampel DB, Dodson P, Osmolska H (2004). Basal ornithischia, chapter fourteen. The dinosauria.

[ref-41] Padian K, Lamm ET (2013). Bone histology of fossil tetrapods.

[ref-42] Paul GS (2010). Dinosaurs: a field guide.

[ref-43] Peng G, Currie PJ, Padian K (1997). Fabrosauridae. Encyclopedia of dinosaurs.

[ref-44] Porro LB, Butler RJ, Barrett PM, Moore-Fay S, Abel RL (2011). New heterodontosaurid specimens from the Lower Jurassic of southern Africa and the early ornithischian dinosaur radiation. Earth and Environmental Science Transactions-Royal Society of Edinburgh.

[ref-45] Porro LB, Witmer LM, Barrett PM (2015). Digital preparation and osteology of the skull of *Lesothosaurus diagnosticus* (Ornithischia: Dinosauria). PeerJ.

[ref-46] Pšenička J, Opluštil S (2013). The epiphytic plants in the fossil record and its example from *in situ* tuff from Pennsylvanian of Radnice Basin (Czech Republic). Bulletin of Geosciences.

[ref-47] Sciscio L, Bordy EM (2016). Palaeoclimatic conditions in the Late Triassic-Early Jurassic of southern Africa: a geochemical assessment of the Elliot Formation. Journal of African Earth Sciences.

[ref-48] Sereno PC (1991). *Lesothosaurus*, “Fabrosaurids”, and the early evolution of ornithischia. Journal of Vertebrate Paleontology.

[ref-49] Sereno PC (2012). Taxonomy, morphology, masticatory function and phylogeny of heterodontosaurid dinosaurs. ZooKeys.

[ref-50] Thulborn RA (1970). The skull of *Fabrosaurus australis*, a Triassic ornithischian dinosaur. Palaeontology.

[ref-51] Thulborn RA (1971). Tooth wear and jaw action in the Triassic ornithischian dinosaur *Fabrosaurus*. Journal of Zoology.

[ref-52] Thulborn RA (1978). Aestivation among ornithopod dinosaurs of the African Trias. Lethaia.

[ref-53] Weishampel DB (1984). Evolution of jaw mechanisms in ornithopod dinosaurs: advances in anatomy, embroyology and cell biology.

[ref-54] Weishampel DB, Norman DB (1989). Vertebrate herbivory in the Mesozoic; jaws, plants, and evolutionary metrics. Geological Society of America Special Papers.

[ref-55] Whitlock JA, Richman JM (2013). Biology of tooth replacement in amniotes. International Journal of Oral Science.

[ref-56] Williams VS (2010). Tooth microwear, diet and feeding in ornithischian dinosaurs. PhD Thesis.

[ref-57] Xia J, Zheng J, Huang D, Tian ZR, Chen L, Zhou Z, Ungar PS, Qian L (2015). New model to explain tooth wear with implications for microwear formation and diet reconstruction. Proceedings of the National Academy of Sciences of the United States of America.

