# Supplementary figures and images for: Digital reconstruction of the mandible of an adult Lesothosaurus diagnosticus with insight into the tooth replacement process and diet
Source: PeerJ. 2017 Mar 1;5:e3054. doi: 10.7717/peerj.3054 (PMC5335715; doi:10.7717/peerj.3054)

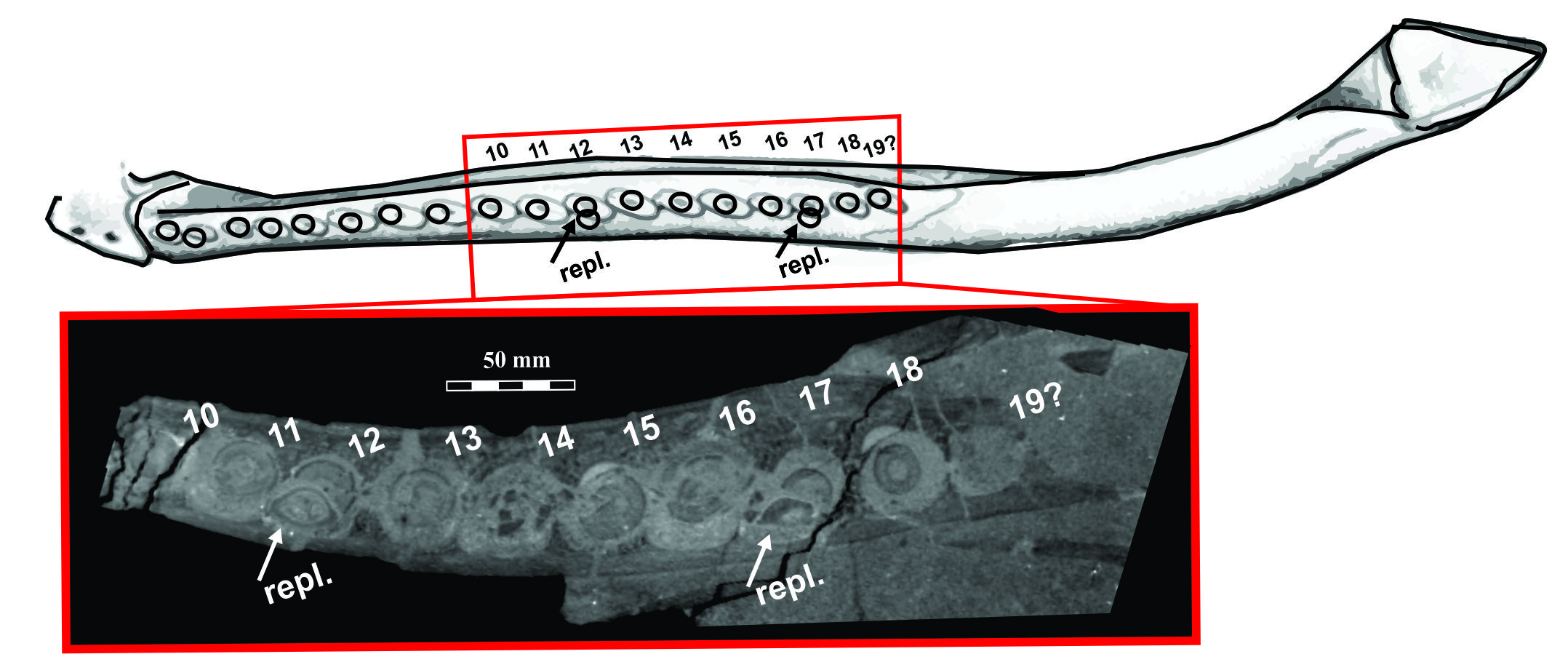

Supplement: Figure S1 — The nine preserved dentary alveoli from tooth positions 10 –18 of BP/1/7853 in comparison to the adapted illustration of Sereno’s (1991: Fig. 13H) reconstruction of the mandible in Lesothosaurus. The ninth tooth socket of BP/1/7853 in the scanned fragment was the 18th in life. This is an estimation used for the sake of ease of discussion. Abbreviation: repl., replacement crown. [file peerj-05-3054-s001.jpg]

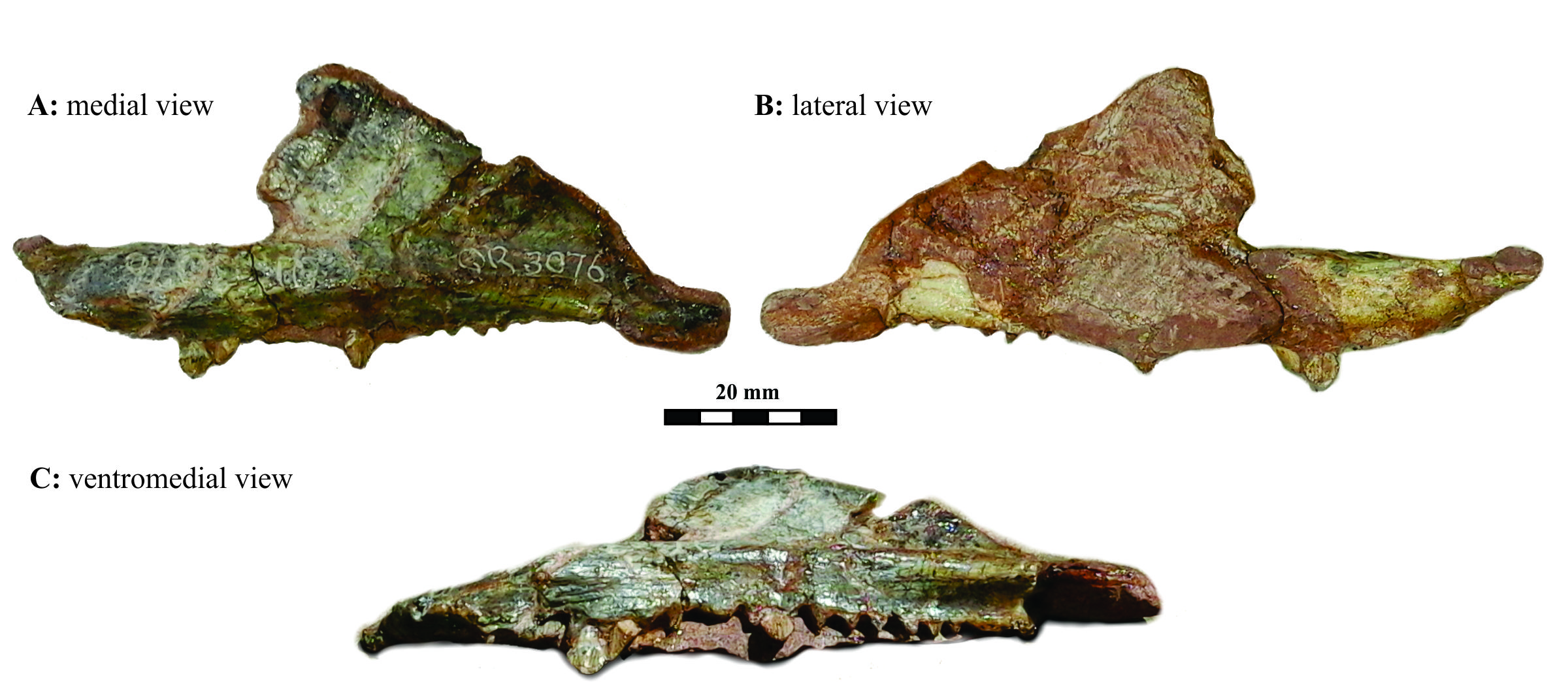

Supplement: Figure S2 [file peerj-05-3054-s002.jpg]
